# Effect of Doxorubicin/Pluronic SP1049C on Tumorigenicity, Aggressiveness, DNA Methylation and Stem Cell Markers in Murine Leukemia

**DOI:** 10.1371/journal.pone.0072238

**Published:** 2013-08-19

**Authors:** Daria Y. Alakhova, Yi Zhao, Shu Li, Alexander V. Kabanov

**Affiliations:** 1 Center for Drug Delivery and Nanomedicine and Department of Pharmaceutical Sciences, College of Pharmacy, University of Nebraska Medical Center, Omaha, Nebraska, United States of America; 2 Laboratory of Chemical Design of Bionanomaterials, Faculty of Chemistry, M.V. Lomonosov Moscow State University, Moscow, Russia; B.C. Cancer Agency, Canada

## Abstract

**Purpose:**

Pluronic block copolymers are potent sensitizers of multidrug resistant cancers. SP1049C, a Pluronic-based micellar formulation of doxorubicin (Dox) has completed Phase II clinical trial and demonstrated safety and efficacy in patients with advanced adenocarcinoma of the esophagus and gastroesophageal junction. This study elucidates the ability of SP1049C to deplete cancer stem cells (CSC) and decrease tumorigenicity of cancer cells *in vivo*.

**Experimental Design:**

P388 murine leukemia ascitic tumor was grown in BDF1 mice. The animals were treated with: (a) saline, (b) Pluronics alone, (c) Dox or (d) SP1049C. The ascitic cancer cells were isolated at different passages and examined for 1) *in vitro* colony formation potential, 2) *in vivo* tumorigenicity and aggressiveness, 3) development of drug resistance and Wnt signaling activation 4) global DNA methylation profiles, and 5) expression of CSC markers.

**Results:**

SP1049C treatment reduced tumor aggressiveness, *in vivo* tumor formation frequency and *in vitro* clonogenic potential of the ascitic cells compared to drug, saline and polymer controls. SP1049C also prevented overexpression of BCRP and activation of Wnt-β-catenin signaling observed with Dox alone. Moreover, SP1049C significantly altered the DNA methylation profiles of the cells. Finally, SP1049C decreased CD133^+^ P388 cells populations, which displayed CSC-like properties and were more tumorigenic compared to CD133^−^ cells.

**Conclusions:**

SP1049C therapy effectively suppresses the tumorigenicity and aggressiveness of P388 cells in a mouse model. This may be due to enhanced activity of SP1049C against CSC and/or altered epigenetic regulation restricting appearance of malignant cancer cell phenotype.

## Introduction

Tumors are complex heterogeneous tissues comprising phenotypically and functionally different cancer cells [1], [2]. One theory suggests that the heterogeneity of tumor cells arises as a result of differentiation of small number of highly tumorigenic “cancer stem cells” (CSC). These cells have high proliferation potential and drive tumor growth and progression. According to CSC model the CSC undergo epigenetic changes similar to normal stem cell differentiation and create a phenotypically diverse nontumorigenic cancer cells with hierarchical organization. These cells were first identified in human myeloid leukemia [3] and then found in many cancers, including breast [4], prostate [5], colon [6], brain [7], and others. The cornerstone of CSC model is that CSC can be phenotypically distinguished from the other tumor cells as they express specific biomarkers characteristic for normal stem cells, such as CD133, ALDH, CD44, etc [8], [9]. However, the biomarker expression does not guarantee that specific cell subpopulation represent or is enriched by CSC. CSC have high tumorigenicity in comparison to other tumor cells, and carry potential to self-renew and differentiate to other tumor cell types. Therefore, in each specific case these cells need to be characterized for tumorigenicity and ability to generate cells of other phenotypes [10]–[13]. CSC are also believed to remain mostly in non-dividing cell cycle state, G_0_, and thus be more resistant to cytotoxic anticancer agents compared to more rapidly dividing cancer cells [14]. Moreover, they often overexpress drug efflux transporters, such as P-glycoprotein (Pgp; ABCB1) and Breast Cancer Resistance Protein (BCRP; ABCG2), which may further assist these cells in escaping conventional chemotherapies [15]. Altogether, CSC can be a source for tumor recurrence, metastasis and drug resistance.

It is also evident that not all cancers have hierarchical organization and follow the CSC model. Alternatively or supplementary to the CSC theory the diversity of tumor cells may arise as a result of the clonal evolution due to stochastic genetic or epigenetic changes [16]. In this case some cancer cells in the tumor may have different tumorigenic potential and, in contrary to CSC model, the number of tumor initiating cells (TIC) may be significant (up to 25%), but the tumors are not hierarchically organized [17], [18]. In other words, all cancer cells have a chance to acquire certain genetic and epigenetic changes to become drug resistant and/or tumorigenic, and tumors can be formed by more tumorigenic cancer cells that are not hierarchically organized [18].

Thus an effective anticancer therapy should be capable of both killing the CSC in hierarchically organized cancers as well as restrict the tumor cell plasticity and epigenetic changes to prevent appearance of tumorigenic and drug resistant cancer cells. One therapeutic modality with such potential has been described and undergone clinical trials. It is SP1049C, a polymeric micelle formulation of Doxorubicin (Dox) with Pluronic block copolymers that has shown in Phase II clinical trial high objective response rates (43%) and increased median survival (10 months) in patients with inoperable metastatic adenocarcinoma of the esophagus and gastroesophageal junction [19]. SP1049C comprises a solution of Dox with a mixture of 0.25% Pluronic L61 and 2% Pluronic F127 in isotonic buffered saline [20]. Pluronics are amphiphilic triblock copolymers of poly(ethylene oxide) (PEO) and poly(propylene oxide) (PPO) with PEO-PPO-PEO structure. Relatively hydrophobic Pluronics (like L61 as well as others with PEO block m.w. of 1,700 to 2,700 kDa and hydrophilic-lipophilic balance (HLB) <18) [20], [21] can sensitize multidrug resistant (MDR) cancer cells resulting in increased cytotoxic activity of Dox, and other drugs by 2–3 orders of magnitude [22], [23]. Thus they target a phenotype similar to that of CSC. Moreover, they prevent development of MDR during prolonged treatments of tumors with chemotherapeutic drugs *in vitro* [24] and *in vivo* [25]. Analysis of gene expression profiles in tumor cells selected upon treatment with Dox/Pluronic formulations suggested that these formulations altered genomic responses of these cells compared to the drug alone [24], [25]. However, the effects of Dox/Pluronic formulations on the tumorigenicity of the resulting cancer cell populations have not been explored. In this study using P388 murine leukemia model we investigate whether SP1049C therapy can 1) decrease tumorigenicity and aggressiveness of cancer cells, 2) prevent overexpression of BCRP and activation of Wnt signaling pathway generally associated with cancer aggressiveness and poor prognosis, 3) affect the DNA methylation patterns indicative of epigenetic gene regulation, and 4) deplete subpopulations of cells with various CSC biomarkers.

## Materials and Methods

### Chemicals and Materials

Thiazolyl Blue Tetrazolium Bromide (MTT, #M5655-1G), Dulbecco’s phosphate buffered saline solution (PBS) were purchased from Sigma-Aldrich (St. Louis, MO). Pluronic P85 (lot # WPYE537B) was kindly provided by BASF Corporation (North Mount Olive, NJ). SP1049C, a formulation of Dox with mixed micelles of Pluronics L61 and F127 was kindly provided by Supratek Pharma. Inc (Montreal, Canada). SP polymers represent the same mixture of Pluronics L61 and F127 without the drug.

### Cell Culture Conditions

Murine leukemia P388 cells were received from the laboratory of Dr. Brian Leyland-Jones at McGill University (Montreal, Canada) [26], [27]. The cells were maintained in RPMI media, supplemented with 10% FBS and 1% penicillin/streptomycin (Gibco) at 37°C and 5% CO_2_.

### Animals

Six-week-old female BDF1 mice (Charles River, Wilmington, MA, USA) were kept five per cage with an air filter cover under light (12 hrs light/dark cycle) and temperature-controlled (22±1°C) environment with food and water *ad libitum*. All manipulations with the animals were performed under a sterilized laminar hood. The animals were treated in accordance to the Principles of Animal Care by National Institutes of Health and Protocol #05-098-01-FC approved by the University of Nebraska Medical Center Institutional Animal Care and Use Committee.

### *In vivo* Cell Transplantation

*In vivo* selection with Dox was done as described previously [25] with some modifications. P388 parental cells (0.5×10^6^/100 µl saline/mouse) were inoculated intraperitoneally (i.p.) to the 6-week-old female BDF1 mice. The mice received three injections i.v. of 1) saline, 2) polymers alone, 3) Dox (2.5 mg/kg) or 4) SP1049C (2.5 mg/kg Dox, 2.25 mg/kg or 0.225 mg/kg polymer mixture) (100 µl/mouse) on days 1, 4 and 7 (Passage 1). (This drug dosing regiment is typical for mouse studies, which commonly report injections of 2.5 mg/kg to 5 mg/kg for Dox every 3rd day [28], [29]). On day 10 the ascitic cells (0.5 mL) were collected with syringe in 5 mL ACK lysis buffer to remove red blood cells, washed twice with PBS and resuspended in serum-free RPMI-1640 media, and used for future analysis. The isolated cells were also used for transplantation to new host animals for the next passage (up to 7 passages, 0.5×10^6^/100 µl saline/mouse i.p.). In in vivo tumorigenicity studies the mice were subcutaneously (s.c.) injected with 200, 1000 or 5000 cells isolated from Passage 1, 4 or 6 animals (4, 5 and 10 animals/group) or with CD133^+^ or CD133^−^ sorted cells (500 and 5000 cells/mice, 4 mice/group). Animals were examined for tumor formation after 2 weeks. The tumor initiating cell frequency was estimated using L-Calc software (www.stemcell.com). The frequency of responding cells is calculated from semilogarithmic plot of the fraction of negative responses as a function of the cell dose. Assuming that one cell is sufficient to produce a tumor, based on Poisson distribution the cell dose yielding 37% negative cultures gives a frequency of cells in the population capable of forming the tumor. For *in vivo* tumor aggressiveness experiment the mice were s.c. inoculated with 5×10^5^ cells isolated from Passage 1 and 4 animals. After the tumors became palpable the tumor volumes were measured using standard calipers and tumor volumes were calculated based on the formula V = 0.5 × L × W^2^.

### Colony Formation Assay

The isolated ascites cells were plated in methylcellulose media (StemCell Technologies, Vancouver, BC, Canada) in 6-well plates (100 cells/well in 1 ml of media, n = 3). The colonies were counted 10 days later.

### Western Blot Analysis

10^6^ tumor cells were lyzed in 100 µl of M-PER protein extraction buffer (Pierce). 10 µg of protein (measured by Bradford assay) was loaded to SDS-PAAG gel and separated via electrophoresis. Then the proteins were transferred to nitrocellulose membrane and probed overnight at 4°C with appropriate monoclonal antibodies. The membrane was washed, incubated with horseradish peroxidase conjugated secondary antibodies of 1 hr at 4°C and detected with enhanced chemiluminescence detection system.

### Genome-wide DNA Methylation Analysis by Methyl-sensitive Cut Counting (MSCC): Construction of the MSCC Library, Data Collection, and Analysis

High-throughput global methylation analysis was performed using the MSCC assay previously described [30]. This procedure uses the methylsensitive restriction endonuclease HpaII, which cuts genomic DNA at all unmethylated CCGG sites. Sample libraries were developed by the University of Nebraska Epigenomics Core facility. Briefly, 2 µg genomic DNA was digested with 20 U of the restriction enzyme HpaII (NEB), and an adapter containing a recognition site for the restriction enzyme MmeI is ligated to digested DNA fragments using T4 DNA ligase (NEB). DNA is ethanol precipitated and nick repaired using 8 U Bst DNA polymerase (NEB). The DNA is then digested with 2 U MmeI to capture the 18 bases adjacent to HpaII sites, and the fragments are then ligated to a second adapter to allow for PCR amplification and final high-throughput sequencing. After appropriate tag size purification using a 10% PAGE gel, the DNA is PCR amplified using BioRad iProof high-fidelity polymerase and quantitative PCR procedures to avoid over-amplification and skewing the production of final tags. Final tags are checked for proper size and concentration using a Bioanalyzer High Sensitivity DNA chip (Agilent) and Qubit Fluorometer (Invitrogen). High-throughput sequencing of library tags was performed by the UNMC Sequencing Core Facility using an Illumina HiSeq 2000 Genome Analyzer. The resulting 18-bp sequence tags are aligned with the mouse genome (mm9) using the short read sequence aligner Bowtie [29]. The library is then matched to all possible unique tags (courtesy of M. Ball, Harvard University, Boston, Massachusetts, USA), and the appropriate gene using perl scripts developed by the UNO/UNMC Genetic Sequence Analysis Facility. Counts of each sequence tag representing a unique unmethylated HpaII site are determined. A change in tag count between control and treatment group indicates a corresponding change in the methylation status of each HpaII CpG. To determine HpaII sites that have a statistically significant change in methylation, comparisons are analyzed using edgeR (a bioconductor package for R programming language), which uses the Bayesian estimation and exact tests based on the negative binomial distribution to make pair wise comparisons between groups [31], [32]. The false discovery rate (FDR) is then determined based on the Benjamini Hochberg method, and an FDR of up to 5% will be considered significant. DNA methylation data have been uploaded to NCBI Gene Expression Omnibus (GEO) under GSE48642 accession number.

### DNA Methylation Data Analysis

The gene promoters that have experienced >20-fold change in methylation after specific in vivo treatment compared to saline control have been identified. Heatmap representation was made using Cluster and TreeView program (http://rana.lbl.gov/EisenSoftware.htm). The area-proportional Venn diagrams were generated using BioVenn online tool (http://www.cmbi.ru.nl/cdd/biovenn/index.php). Functional identification of gene networks was performed using Ingenuity Pathway Analysis program (Ingenuity Systems, CA, USA).

### Aldehyde Dehydrogenase (ALDH) Activity Measurement

To assess ALDH activity of the different cell lines, the ALDEFLUOR® assay kit (StemCell Technologies, Vancouver, BC, Canada) was used. Ascites cells were washed with ACK buffer and analyzed according to manufacturer’s protocol.

### Cell Sorting

CD133^+^ and CD133^−^ cells were isolated from Passage 4 or Passage 5 saline treated mice by magnetic sorting. For this the ascitic cells were labeled with magnetic bead-conjugated anti CD133 monoclonal antibodies according to manufacturer’s protocol (Myltenyi Biotech, order No 130-092-333) and separated by autoMACS™ Separator. ALDH^+^ and ALDH^−^ cells were isolated from Passage 4 saline treated mice by FACS. The ALDH^+^ cells were identified with ALDEFLUOR® assay kit and CD34^+^/CD38^−^ cells were identified using fluorescently labeled antibodies as described above. For each sorting 10–40×10^6^ cells were used. After sorting the cells were labeled with trypan blue and live and dead cells were counted.

### Fluorescence Activated Cell Sorting (FACS) Analysis

Ascites cells were washed with ACK lysis buffer to lyse the blood cells and resuspended in FBS-free RPMI medium. For CD34/CD38 and CD133 expression profiles 10^6^ were incubated on ice with Alexa 647 anti CD34/Alexa 488 anti CD38 antibodies or with PE-anti CD133 (Biolegend) for 30 min. After the incubation the cells were washed with ice-cold PBS, centrifuged, resuspended in 0.5 ml PBS and analyzed by FACS.

### Cytotoxicity Studies

Dox and SP1049C cytotoxicity was evaluated in parental P388 (that were not passaged *in vivo*), CD133^+^, CD133^−^, CD34^+^/CD38^−^, ALDH^+^, ALDH^−^ and unsorted cells, isolated from saline treated mice from Passage 4 or 5. 5000 cells/well were plated in 96-well plate in RPMI media. The following day the cells were treated with different concentrations of Dox or Dox with 0.001% SP1049C polymer mixture. 72 or 144 hrs later the cell viability was evaluated using CCK-8 assay. The viability was calculated as % of untreated control. Each drug concentration was tested in 8 wells. Data are presented as mean ± SD.

### Rhodamine 123 (Rh123) Uptake Studies

Rh123 uptake studies were done as described previously [25]. Briefly, 10^6^ of CD133^+^ or CD133^−^ cells were pre-incubated for 30 min in assay buffer at 37°C (122 mM sodium chloride, 25 mM sodium bicarbonate, 10 mM glucose, 10 mM HEPES, 3 mM potassium chloride, 1.2 mM magnesium sulfate, 1.4 mM calcium chloride, and 0.4 mM potassium phosphate dibasic, pH 7.4) and then treated with 3.2 µM Rh123 for 60 min. After that the cells were washed 3 times with ice-cold PBS and solubilized with 1% Triton X100. Rh123 fluorescence was measured using Molecular Devices Spectramax M5 at λ_ex_ 505 nm and λ_em_ 540 nm. The fluorescence was normalized over protein content, determined by BCA assay. Each measurement was repeated in 8 wells.

### Statistical Analysis

In FACS analysis of cell subpopulations (CD133^+^, CD34^+^/CD38^−^, ALDH^+^) analysis each data point represents average of 3 measurements ± SD. The data were analyzed using Student’ t-test.

## Results

### SP1049C Decreases Colony Formation Potential of Cancer Cells

**Figure 1:**
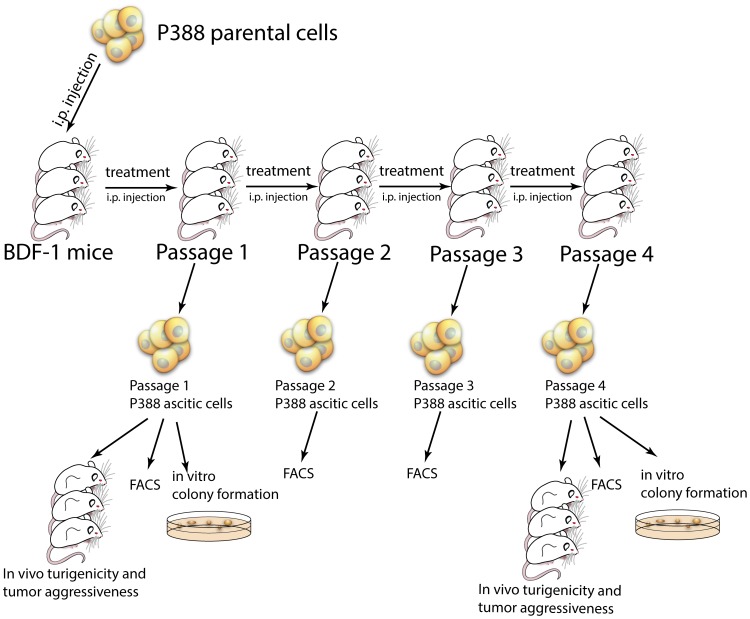
*In vivo* selection model. 5×10^5^ P388 cells/mice were inoculated i.p to 6-week-old BDF1 mice. The mice *(n = 4/group)* were injected on days 1, 4 and 7 i.v with (a) saline, (b) polymer mixture, (c) doxorubicin or (d) SP1049C. On day 10 the ascites were collected for further analysis. 5×10^5^ cells/mice were transferred to new host animals for the next treatment passage. Ascites from Passage 1 and 4 were used for tumor aggressiveness and tumorigenicity studies in s.c. tumor model.

**Figure 2:**
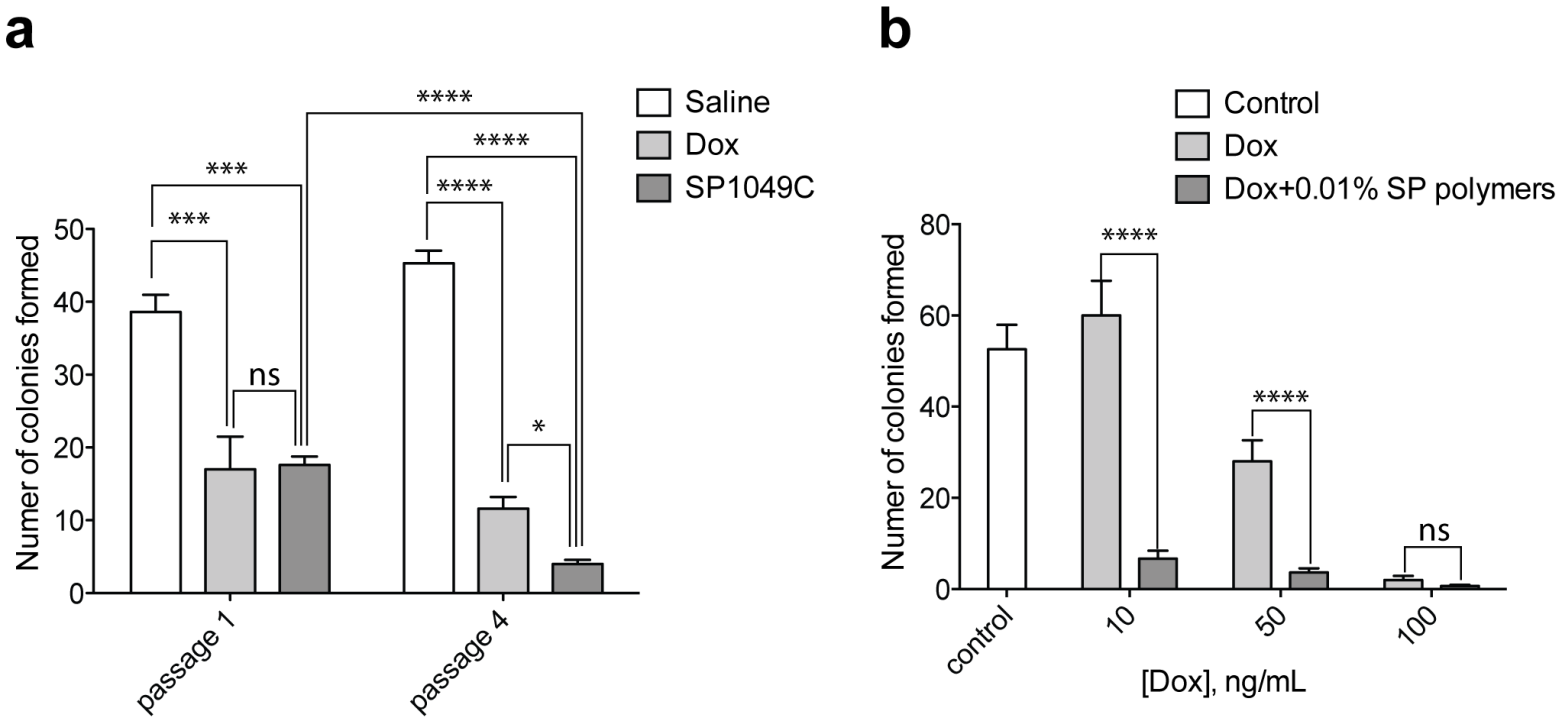
Number of colonies formed by (a) P388 ascitic cells after different chemotherapy regimens (saline, Dox (2.5 mg/kg) and SP1049C (2.5 mg/kg Dox, 2.25 mg/kg polymer mixture) and (b) P388 ascitic cells after 2 hr pretreatment with Dox (10, 50 and 100 ng/mL) with or without 0.1% SP polymers (100 cells seeded/well in 6-well plates, methyl cellulose media, assayed on day 10–14). Data presented as average ± SD (n = 3). *p<0.05; ***p<0.001; ****p<0.0001.

The ability to form colonies *in vitro* in semi-solid medium supplemented with appropriate growth factors and other nutrients is regarded as one of the major properties of trumorigenic cancer cells [33] and is considered a surrogate for *in vivo* transplantation. To evaluate the effect of different in vivo treatment regiments (saline, Dox and SP1049C) on colony formation potential P388 murine leukemia cells were selected *in vivo* as described in materials and methods (**Figure 1**). In this design in order to prolong the *in vivo* exposure of the cancer cells to the treatments the animals with ascitic tumors were treated with the drug three times and then the cancer cells were transferred to a new host animal (new “passage”) to continue treatment. This was repeated up to 6 passages. This protocol ensured that in each given passage the animals did not become moribund due to growing ascitic cells. For example, it was reported that without treatment the BDF1 mouse with P388 ascitic cells has a mean survival time of approximately 10.3 days [34]. Our prior study has shown that using similar protocol the drug treatments extended the survival and well-being of animals with the P388 ascitic cells [25]. In this study however, the animals treated with Dox alone developed drug resistance and exhibited elevated levels of Pgp after approximately 9 passages, *which was abolished in Dox/Pluronic cohorts* [25]. So we essentially focused on the events preceding development of MDR in the majority of the cell population. The treatment groups included 1) saline, 2) polymers alone, 3) Dox and 4) SP1049C. These treatments were administered i.v. on days 1, 4, and 7. On day 10 the ascitic fluids were collected for further analysis and at the same time transferred to new hosts. Notably, at the end of each passage SP1049C treated animals have shown greater vital signs (mobility, activity) compared to Dox alone and especially control and polymer alone groups. Yet in all groups the animals did not become moribund. In SP1049C and Dox treatment groups in all passages the ascitic fluids appeared to be smaller than those in the control and polymer alone groups. This was most pronounced in SP1049C cohorts of the later passages (e.g. Passage 4). However, in each case we were able to collect sufficient amount of cells. The ascitic cells from Passage 1 and Passage 4 animals were plated in methylcellulose media for colony formation. In Passage 1 the SP1049C effect was comparable to Dox alone, however, by Passage 4 the ascitic cells from SP1049C treated mice formed significantly lower number of colonies compared to both saline and Dox groups (**Fig. 2a**). Notably, the ability of cells to form colonies after *in vivo* treatment with SP1049C decreased from Passage 1 (17.6±2) to Passage 4 (4±1). We next evaluated the effect of *in vitro* treatment of cells with Dox and SP1049C on the colony formation potential. For this purpose the cells isolated from Passage 4 saline treated mice were pre-treated for 2 hr with 10, 50 and 100 ng/mL Dox with or without 0.1% SP polymers, washed and plated in methylcellulose media (**Fig. 2b**). The cell viability was confirmed before plating using trypan blue staining and only live cells were accounted for the appropriate dilution. 10 ng/mL of Dox alone didn’t affect the colony formation compared to control (60±13.22 and 52.6±9.3 respectively). On the contrary 10 ng/mL with 0.1% SP polymers decreased the number of colonies formed from 52.6±9.3 to 6.66±3. Comparable effect of Dox alone pretreatment was achieved only at 100 ng/mL. Overall, these findings provide strong evidence that SP1049C suppresses the colony formation potential of tumor cells after both *in vivo* and *in vitro* treatments.

### SP1049C Decreases Tumorigenicity and Aggressiveness of P388 Cells *in vivo*

The ability of isolated ascites cells to form tumors *in vivo* was evaluated in limiting dilution assay. The mice were s.c. inoculated with three different doses of cells: 200, 1000 and 5000, isolated from ascites of mice from Passages 1 and 4, and the tumor formation have been monitored for two months (**Table 1**). We found that in both Passages 1 and 4 the tumorigenic potential of the ascitic cells, isolated from SP1049C treated mice was lower compared to Dox and saline groups. The results for the SP polymers alone group were similar to those of the saline control group (**Table 1**). **Table 2** shows the frequencies of tumor forming cells in Passages 1 and 4 calculated using L-Calc software (StemCell). The frequency of responding cells is calculated from semilogarithmic plot of the fraction of negative responses as a function of the cell dose. Assuming that one cell is sufficient to produce a tumor, based on Poisson distribution the cell dose yielding 37% negative cultures gives a frequency of cells in the population capable of forming the tumor. In SP1049C treatment group already in Passage 1 the cells exhibited a trend for lower tumorigenicity compared to saline and Dox groups. Specifically, only 1 cell out of 2,260 was able to form the tumor in SP1049C group, while for saline, SP polymers alone and Dox groups this frequency was 1 out of 1,109, 1 out of 1,608 and 1 out of 380 cells respectively. Furthermore, by Passage 4 the TIC frequency in SP1049C treatment group decreased almost twice compared to passage 1 and was estimated as 1 in 4201 cells, while in saline and Dox groups it was significantly higher - 1 in 336 and 1 in 778 respectively (**Table 2**). Notably, since in SP1049C groups the volume of the ascitic fluid was less compared to other treatment and control groups the absolute numbers of TIC after SP1049C treatment may be even further decreased that it was evident by the frequency numbers, especially in the later passages. Both in passage 1 and 4 the TIC frequencies in SP polymers alone groups were not significantly different from those of the saline control group (**Table 2**).

**Table 1:** Limiting dilution assay for tumor formation frequency.

Cell dose	Saline		SP polymers alone		Dox		SP1049C	
	P1	P4	P1	P4	P1	P4	P1	P4
200	1/4	4/5	1/4	3/5	1/4	2/5	1/4	0/5
1000	2/4	4/5	1/4	4/5	4/4	3/5	2/4	2/5
5000	4/4	5/5	4/4	4/5	4/4	5/5	4/4	3/5

The cells were isolated from ascites of mice from Passage 1 (P1) and Passage 4 (P4) treated with saline, SP polymers alone (2.25 mg/kg), Dox (2.5 mg/kg) or SP1049C (2.5 mg/kg Dox, 2.25 mg/kg polymer mixture) and s.c. inoculated to new mice at three different doses.

**Table 2:** Estimated frequencies of tumor initiating cells in ascites from Passages 1 and 4 in Saline, Dox and SP1049C treatment groups.

Passage #	Saline	SP polymersalone	Dox	SP1049C
P1	1 in 1109	1 in 1608	1 in 380	1 in 2260*
P4	1 in 336	1 in 422	1 in 778	1 in 4201*

The frequencies were calculated using L-Calc software (http://www.stemcell.com/) based on tumor formation frequency data. Statistical comparisons are made pair wise between the groups within each passage *p<0.05.

**Figure 3:**
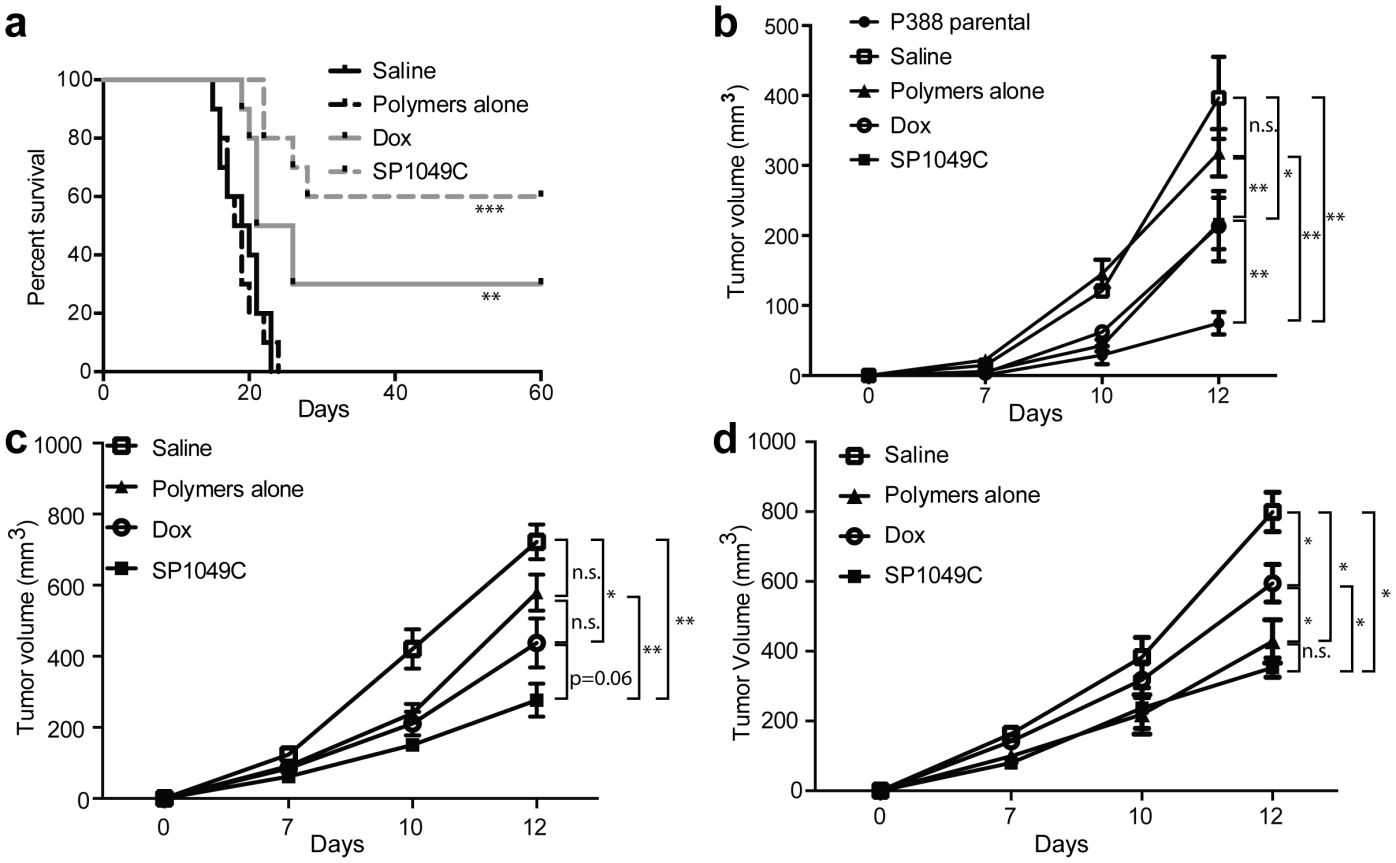
SP1049C treatment decreases tumor aggressiveness. (**a**) Lifespan of animals injected with (5×10^3^ cells) cells from Passage 6 (n = 10/group); (**b, c, d**) Rate of s.c. tumor growth of P388 ascitic cells after different chemotherapy treatment regimens (5×10^5^ cells/mice from (**b**) Passage 1**,** (**c**) Passage 4 and (**d**) Passage 7 were s.c inoculated and tumor volumes measured every other day (n = 5/group)). Data presented as average ± SEM. (**a**) Survival curves were compared to the saline group using the log-rank (Mantel-Cox) test; (**b,c,d**) tumor volumes comparisons are presented for day 12 data point; the comparisons were made using Student’s t-test. *p<0.05, **p<0.01, ***p<0.001, n.s. – not significant.

In a second set of experiments the mice were s.c. inoculated with 5000 cells isolated from Passage 1 and 6 ascites (**Table S1**). The ascites bearing mice in this selection of experiments were treated with 10 times lower concentration of SP polymers while the Dox concentration was kept the same. Even at this low dose of SP polymers by Passage 6 SP1049C treatment reduced the tumorigenicity of the ascitic cells compared to Dox alone and control groups. Specifically, only 4 out of 10 mice formed the tumors when injected with the cells isolated from the SP1049C treated animals. In contrast, the tumors were formed in 7 out of 10 animals injected with the cells from Dox group, and in 10 out of 10 animals injected with the cells from saline and polymers alone groups. We further monitored the survival of these animals (**Fig. 3a**). The mice inoculated with cells from SP1049C group had the longest life span and the highest survival rate compared to the mice inoculated with cells from Dox, saline and polymers alone groups.

Next we evaluated the *in vivo* tumor aggressiveness by measuring the rate of the tumor growth. In this experiment the mice were s.c. inoculated with 5×10^5^ cells from Passage 1, Passage 4 and Passage 7 ascites (**Fig. 3b, 3c, 3d**). In Passage 1 there was no difference between saline and SP polymers alone and between Dox and SP1049C groups, although both drug treatments resulted in some decrease of the tumor aggressiveness. Interestingly, the P388 parental cells (that were not passaged in vivo) produced the slowest growing tumors (**Fig. 3b**). Among cells isolated from Passage 4 those of the saline group formed the most aggressive tumors (**Fig. 3c**, the images of the tumors in this panel are presented in **Fig. S1a**). In contrast, in SP1049C group the tumor aggressiveness was significantly decreased compared to saline and polymers alone groups (**Fig. 3c**). SP1049C group also showed slower tumor growth compared to Dox group albeit the significance level was not sufficient p = 0.06. In another experiment using this passage at a later time point (day 14) the tumor growth in the SP1049C group was significantly slower than both in the saline control and Dox alone groups (**Fig. S1b**). In the case of cells isolated from passage 7 the tumor growth in SP1049C group was also significantly slower in the saline and Dox alone controls (**Fig. 3c**). Interestingly in this passage the SP-polymer group also revealed significant decrease in the tumor growth that was not evident in the earlier passages.

### SP1049C Prevents BCRP Expression and Suppresses Wnt Signaling Activation

**Figure 4:**
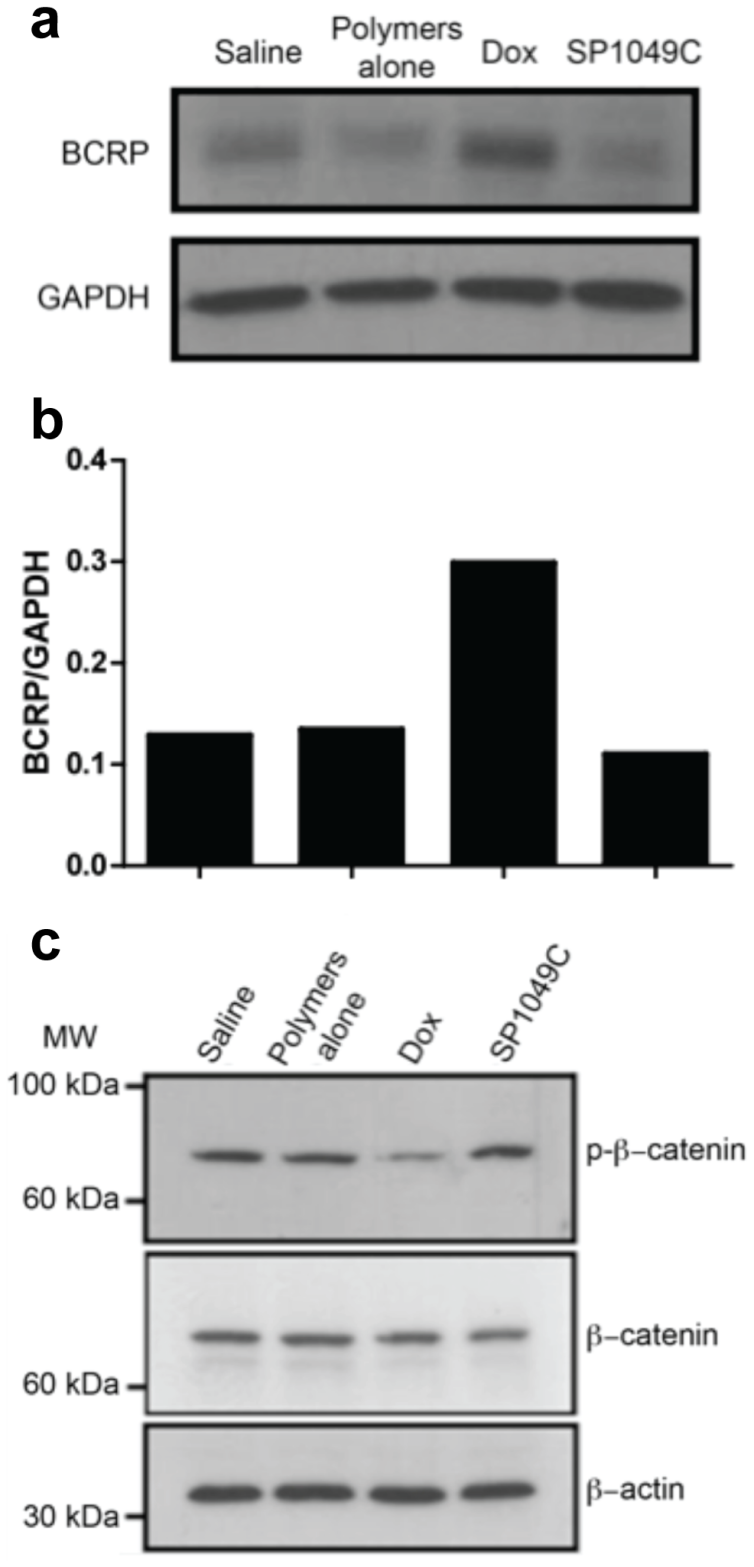
BCRP expression in ascites cells in Passage 6 and Wnt signaling activation in tumor cells from Passage 5. (**a**) BCRP expression levels, (**b**) densitometry analysis of BCRP western blot, (**c**) phospho-β-catenin and β-catenin protein levels. β-actin was used as protein loading control.

Using a similar *in vivo* ascitic model we have previously shown that Pluronic prevents the development of MDR and Pgp overexpression in P388 cells by Passages 9 to 10 [25]. In the present study we examined the BCRP expression in ascitic cells in Passage 6. By this Passage there was a detectable level of BCRP even in the saline treatment group, although the parental cells did not exhibit BCRP at all (**Fig. 4a**). As seen in **Fig. 4**, Dox alone treatment resulted in substantially increased BCRP expression compared to the saline group. In contrast, in the SP1049C group the BCRP levels did not increase. No changes in BCRP expression compared to the control were observed in the polymers alone group.

Next we evaluated activation of Wnt/β-catenin signaling in ascites cells upon different treatments. To this end, the amounts of phosphorylated and dephosphorylated β-catenin were determined in the cell lysates. As shown in **Fig. 4c**, Dox treatment has induced dephosphrylation of β-catenin in Passage 5, suggesting activation of Wnt signaling. In contrast, in the SP1049C group the levels of phospho-β-catenin remained unchanged compared to these in the control or polymer mixture groups. β-catenin is a transcription factor, which in un-activated cells localizes in the cytoplasm. When Wnt signaling is activated β-catenin gets dephosphorylated and translocate to the nucleus where it activates target genes [35].

### SP1049 Alters DNA Methylation Profile of P388 Cells *in vivo*

**Figure 5:**
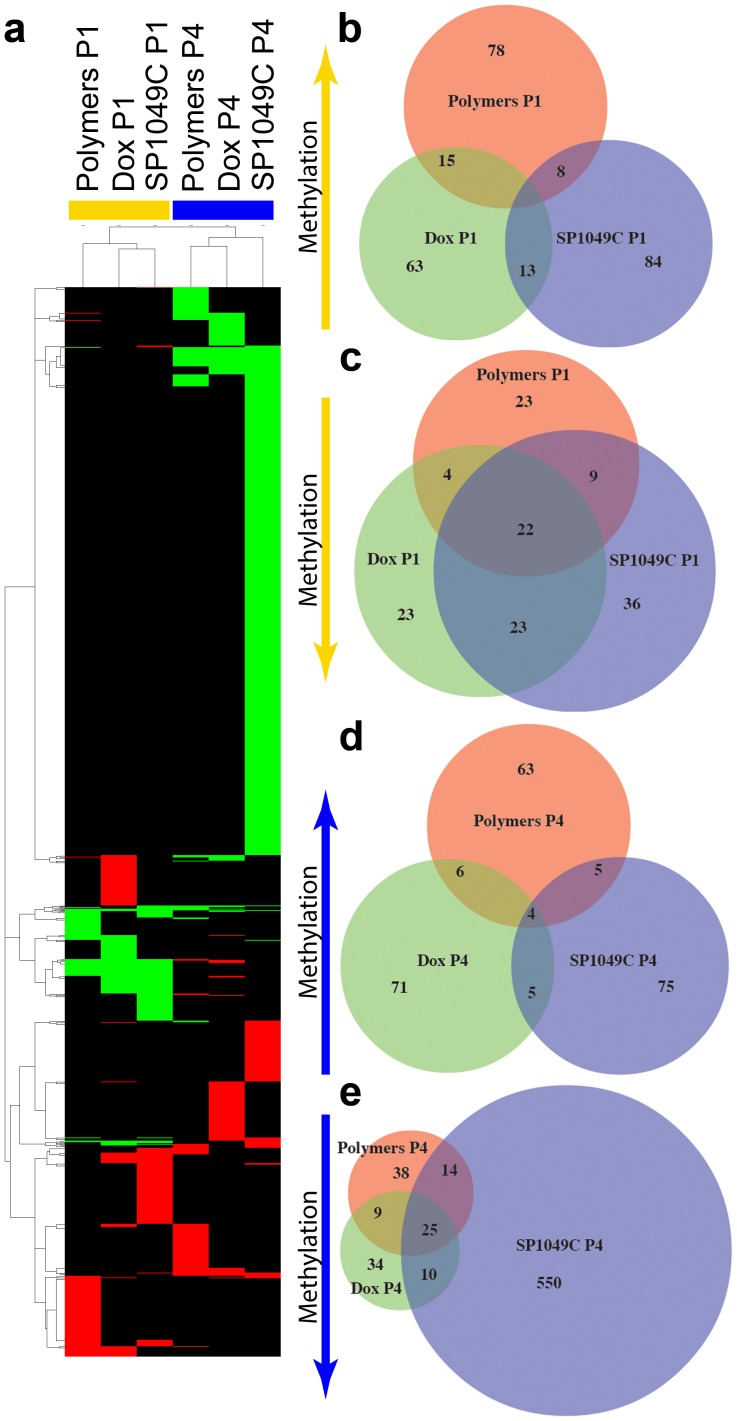
Differentially methylated gene promoters in P388 ascitic cells isolated from Passages 1 (P1) and 4 (P4). The genes that experienced >20 fold change in methylation after different *in vivo* treatments (SP Polymers alone, 2.25 mg/kg, Dox, 2.5 mg/kg and SP1049C, 2.5 mg/kg Dox, 2.25 mg/kg SP Polymers) (compared to saline control of corresponding passage) are shown. (**a**) Heatmap representation of differentially methylated genes in P1 (yellow bar above the heatmap) and P4 (blue line): red and green correspond to the hyper- and hypomethylation, respectively, whereas black corresponds to no difference between the treatment and the saline control. (**b,c,d,e**) Venn diagrams of the number of hyper- ((**b**) – P1, (**d**) – P4) and hypomethylated ((**c**) – P1, (**e**) – P4) genes. Numbers indicate the number of genes in each sector.

Epigenome is represented by heritable yet reversible chemical changes in DNA and histones that regulate gene expression. During carcinogenesis along with genetic mutations cells undergo drastic changes in DNA methylation patterns [36]. Therefore, we studied the effect of SP polymers, DOX and SP1049C treatments on global DNA methylation status during the *in vivo* treatment course. We found that significant number of gene promoters was affected in all treatment groups, therefore to narrow down the results we identified the gene promoters, that have experienced >20 fold change in methylation compared to saline control from corresponding passage (**Fig. 5** **)**. Full list of identified genes is shown in **Table S3**. In Passage 1 total of 165, 179 and 205 gene promoters were affected in SP polymers, DOX and SP1049C treatment groups respectively, while in Passage 4 total of 177, 180 and 737 gene promoters were affected in the corresponding treatment groups. The selected genes are shown as a heatmap in **Fig. 5a**. Notably, DNA methylation and demethylation events occurred at relatively same frequency in SP polymers and DOX treatment groups in both passages, whereas in SP1049C group promoter demethylation significantly increased in Passage 4 and number of methylated promoters remained similar to Passage 1. Venn analysis of the identified gene promoters showed that methylated genes in both Passages exhibit very small overlap between the treatment groups (**Fig. 5b and 5d**). Demethylated genes on the other hand overlap to a higher extent (**Fig. 5c and 5e**). Overall SP1049C treatment resulted in strongest effect on DNA methylation status and to significantly higher extent resulted in hypomethylation of gene promoters compared to hypermethylation.

We further analyzed the biological relevance of the affected genes using Ingenuity Pathway Analysis (IPA) (Ingenuity Systems, CA, USA) (**Fig. S2**). Top five biological functions (**Fig. S2a and S2b**) and canonical pathways (**Fig. S2c and S2d**) were identified in each group based on significance. The most significant changes were observed in biofunctions in Passage 4 SP1049C treatment group (**Fig. S2b**). Interestingly, the affected most significant canonical pathways were completely different for each treatment group and passage, suggesting that each treatment individually had distinct effect on DNA methylation in the cells, and only SP polymers alone and SP1049C groups have shown partial overlap in affected biofunctions in both passages. Furthermore, in Dox group from Passage 4 top affected biofunctions related to cellular and drug metabolism and small molecule transport, while in SP1049C group the affected biofunctions were of completely different character and mostly related to cellular development, growth, differentiation, function and maintenance. Noteworthy, DNA demethylation had significantly stronger impact in these biofunctions (**Fig. S2b**). Moreover, when analyzing the top canonical pathways affected by the three treatments we found that in SP1049C group in Passage 4 three out of five top canonical pathways regulate embryonic stem cell differentiation, pluripotency and transcriptional regulatory network. Importantly, all genes significantly affected in these pathways were demethylated (**Fig. S2d**).

### Effect of SP1049C on CD133^+^, ALDH^+^ and CD34^+^/CD38^−^ Cell Populations in P388 Ascites

**Figure 6:**
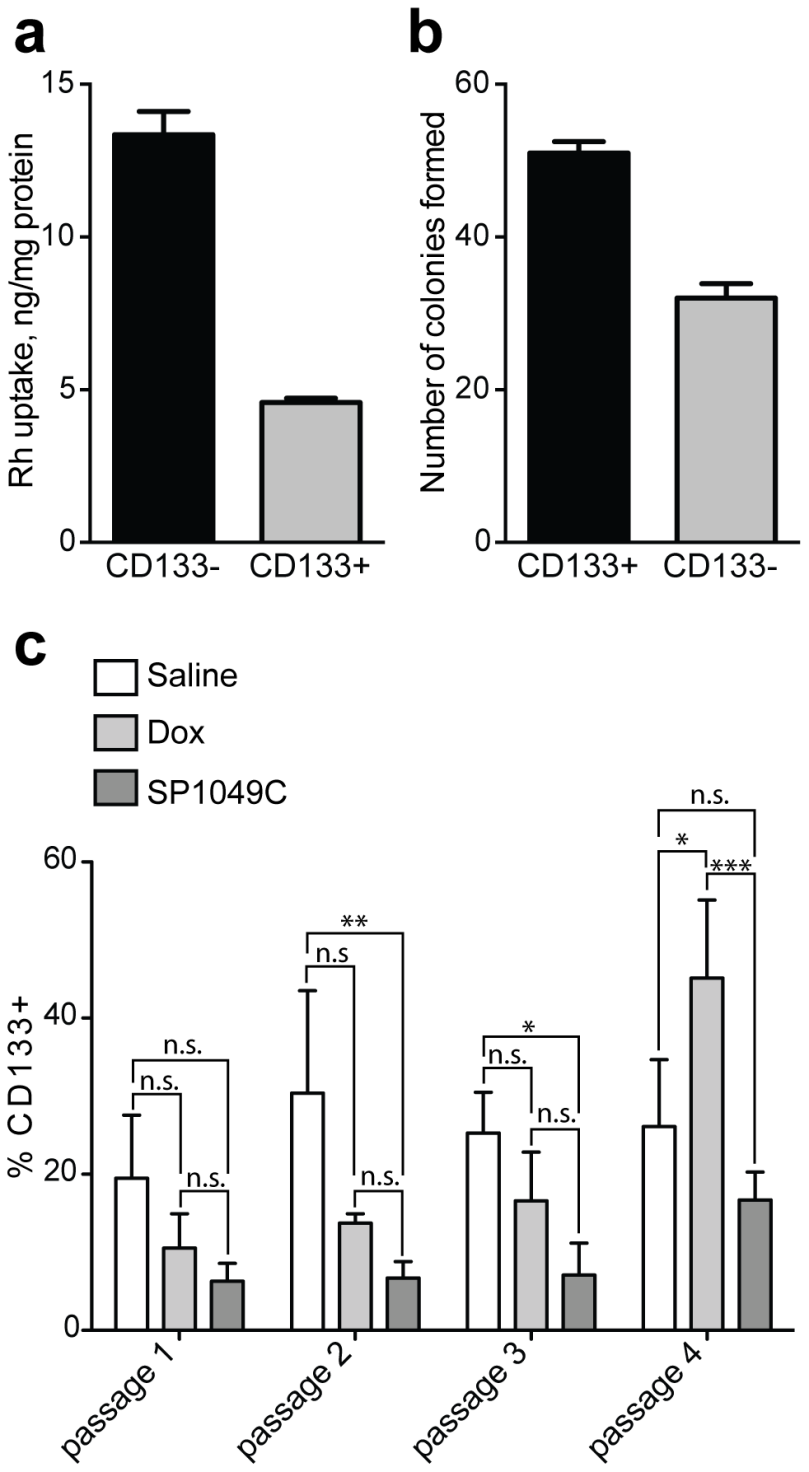
CD133^+^ cell subpopulation in ascites tumors during *in vivo* selection of P388 cells. (**a**) Exclusion of Rh123 from CD133^+^ cells isolated by magnetic sorting from Passage 4 saline treated mice; (**b**) number of colonies formed by CD133^+^ and CD133^−^ cells isolated from Passage 5 saline treated mice (100 cells seeded/well in 6-well plates, methyl cellulose media, assayed on day 10–14); (**c**) fractions of CD133^+^ cells after different chemotherapy regimens in Passages 1–4. Treatments: 1) saline, 2) Dox (2.5 mg/kg) or 3) SP1049C (2.5 mg/kg Dox, 2.25 mg/kg). Data presented as average ((a) n = 8, (b) n = 3, (c) n = 3) ± SD. *p<0.05, **p<0.01, ***p<0.001, n.s-not significant.

Major characteristic of CSC is that they have specific phenotypic profile distinct from other tumor cells. Therefore, we further evaluated the expression of several markers, previously reported to be associated with CSC. CD133 glycoprotein is a specific stem cell marker, which was implicated in CSC in many cancers [37], [38]. However, in some cancers CD133 expression is not unique for CSC [39] [40]. Thus, to validate this marker in our cancer model we isolated CD133^+^ and CD133^−^ cells subpopulations from saline treated mice from Passage 4 using magnetic sorting as described in materials and methods. Comparison of these subpopulations suggests that CD133^+^ cells have 1) increased level of functional Pgp efflux system (as followed from exclusion of a Pgp substrate, **Fig. 6a**), 2) increased resistance with respect to Dox (**Table 3**), 3) increased colony formation potential *in vitro* (**Fig. 6b**) and 4) increased tumorigenicity *in vivo* (as assayed by tumor frequency formation after s.c. inoculation of cells in mice, **Table 4**). Therefore, CD133^+^ cells display functional CSC-like properties and are more tumorigenic and chemoresistant compared to CD133^−^ cells.

**Table 3:** Dox cytotoxicity in P388 parental cells (that were not passaged in vivo), CD133^+^ and CD133^−^ cells isolated from Passage 4 saline treated mice using magnetic sorting.

Treatment	Dox IC_50_, ng/mL		
	P388	CD133^+^	CD133^−^
Dox	17.5	37.24	18.41

Dox cytotoxicity was evaluated after 48 h incubation.

**Table 4:** In vivo tumor formation frequency of CD133+ and CD133- cells.

Cell dose	CD133^+^	CD133^−^
500	4/4	0/4
5000	4/4	1/4

Magnetically sorted CD133+ and CD133- cells were s.c. inoculated to BDF1 mice at two cell doses. The animals were examined for tumor formation after 2 weeks.

Next we evaluated the effects of different treatments on the expression of CD133 in ascitic P388 cells, isolated from different passages **(** **Figure 1**). In Passage 1 there was no significant effect of treatment on CD133^+^ cell population. In Passages 2 and 3 we observed depletion of CD133^+^ cells in SP1049C group compared to saline. Dox alone treatment did not have this effect and the difference between the Dox and saline groups was not significant (**Fig. 4c**). At Passage 4 the Dox group exhibited significant increase in CD133^+^ cells subpopulation compared to control, while SP1049C at Passage 4 significantly depleted CD133^+^ cell subpopulation in comparison to Dox.

**Figure 7:**
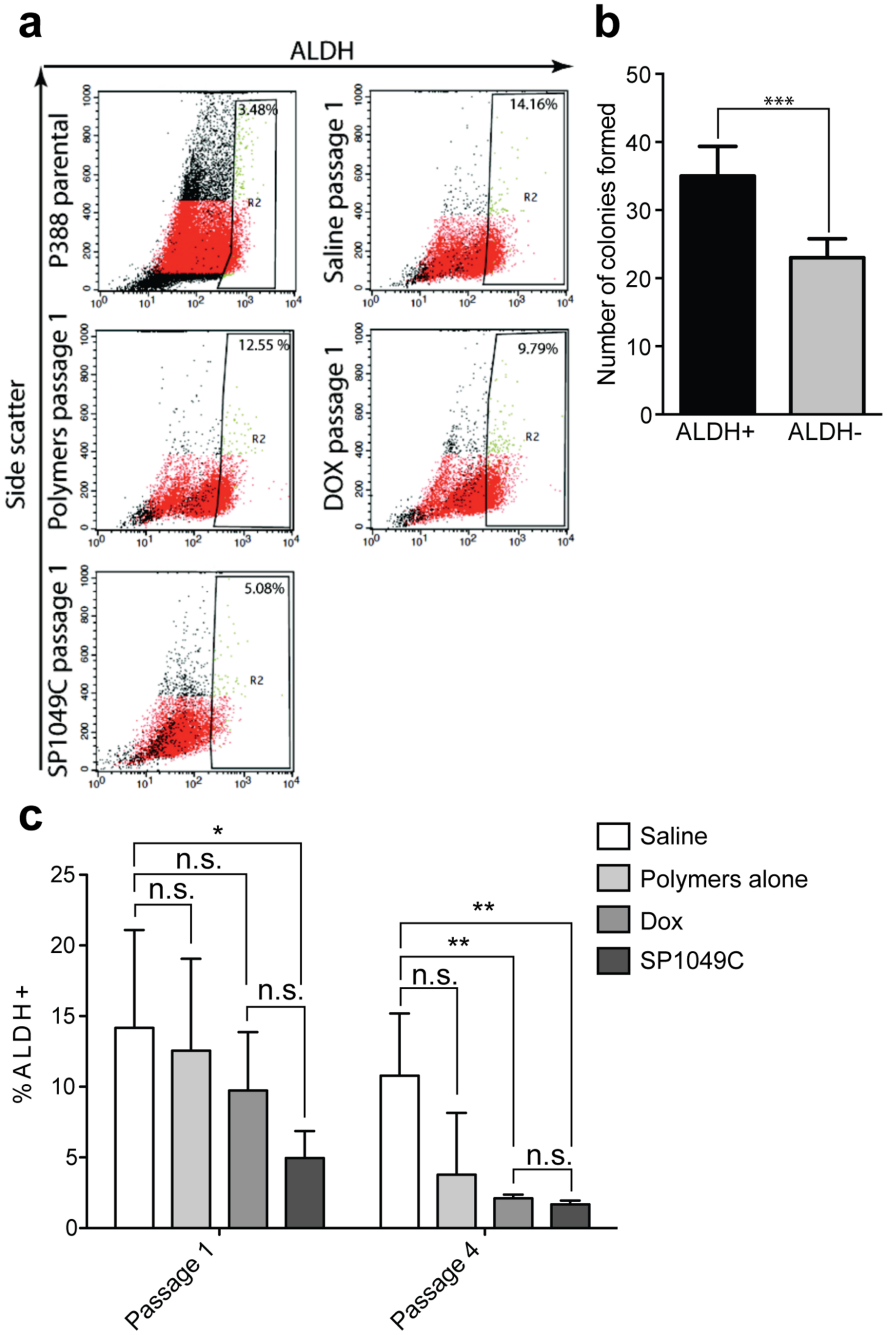
Depletion of ALDH^+^ cells during *in vivo* selection. Ascite cells were collected, washed with ACK buffer and analyzed for ALDH activity using ALDEFLUOR assay according to manufacturers protocol. (**a**) ALDEFLUOR assay of P388 parental cells and ascitic cells from Passage 1; (**b**) number of colonies formed by ALDH^+^ and ALDH^−^ cells isolated from Passage 4 saline treated mice (100 cells seeded/well in 6-well plates in methyl cellulose media, assayed on day 10–14); (**c**) fractions of ALDH^+^ cells after different chemotherapy regimens and Passages. Treatments: 1) saline, 2) SP polymers alone (0.225 mg/kg), 3) dox (2.5 mg/kg) or 4) SP1049C (2.5 mg/kg dox, 0.225 mg/kg polymer mixture). Data presented as average ((b, c) n = 3) ± SD. *p<0.05, **p<0.01, n.s. - not significant.

ALDH is another stem cell marker, which is often highly overexpressed in normal and cancer precursor cells [8], [41], [42]. To validate this marker in the present tumor model the P388 ascitic cells were passaged in mice as described above followed by isolation of the ALDH^+^ and ALDH^−^ cell sub-populations by FACS using ALDEFLUOR assay (**Fig. 7a**). Comparison of these subpopulations suggested that ALDH^+^ cells have increased *in vitro* drug resistance (**Fig. S3)** and increased colony formation potential (**Fig. 7b**). Next it was demonstrated that both Dox and SP1049C treatments depleted ALDH^+^ cells (**Fig. 7c**). Already in Passage 1 the significant decrease in ALDH^+^ cell subpopulation after SP1049C treatments compared to saline control was observed. By Passage 4 both Dox and SP1049C significantly depleted ALDH^+^ cells, however, there was no difference between the two treatments.

Finally, CD34^+^/CD38^−^ cells have been identified as leukemia initiating cells in human acute myeloid leukemia [3], [43]. They were shown to be more resistant to Daunorubicin and have increased expression of multidrug resistance proteins (MRP/LRP) compared to CD34^+^/CD38^+^. However, in our model of murine leukemia these markers were neither associated with the increased drug resistance (**Table S2**) nor increased colony formation potential (**Fig. S4a**). Analysis of the effects of different treatments on these subpopulations suggests that albeit both Dox and SP1049C significantly depleted CD34^+^/CD38^−^ cells at Passage 1 compared to control, the differences between treatment and control groups were not significant at Passages 4 (**Fig. S4b**).

## Discussion

The tumorigenicity and aggressiveness of cancer cells are critical factors for patients’ survival. This study clearly demonstrated that treatment with SP1049C of animals with ascitic murine leukemia decreased the colony formation ability of the cancer cells, as well as the frequency of tumor formation and tumor growth rates following re-inoculation of these cells in mice. Moreover, the lifespan of the tumor-bearing animals was also increased in SP1049C treated groups. Taken together these findings demonstrate that SP1049C effectively suppresses the tumorigenicity and aggressiveness of cancer cells. This may explain high activity of SP1049C exhibited in a Phase II trial in patients with advanced esophageal cancer [19], although the difference with our murine leukemia model should be clearly recognized. Notably, in preclinical toxicology studies in rodent and non-rodent species it was shown that SP1049C has similar toxicity profiles as Dox alone [44]. Moreover, the toxicity profile of SP1049C was similar to that of Dox in the clinical trials [45].

As shown in this work Dox alone and SP1049C exhibited differential effect on the Wnt/β-catenin signaling pathway in leukemia cells. The activation of this pathway was observed with the drug alone. However, SP1049C treatment did not affect this pathway, since the amount of phospho-β-catenin in leukemia cells remained the same as in the control group. The Wnt/β-catenin signaling pathway has been shown to play a pivotal role in self-renewal of normal stem cells and embryogenesis [46]. Furthermore, it is involved in malignant transformation, and associated with tumor aggressiveness, metastasis and poor clinical outcome [47]–[49]. Chronic upregulation of Wnt signaling has been observed in several cancers and was also reported to play an important role in CSC [46]. Therefore, this observation is generally consistent with the decrease in tumorigenicity and aggressiveness of P388 cancer cells upon SP1049C treatments, as well as with the increase in tumorigenicity and aggressiveness of cancer cells upon Dox alone treatment [34], [50]. However, the functional significance of the observed changes in Wnt/β-catenin signaling pathway has not been demonstrated in this study and remains to be further explored.

SP1049C treatment also prevented the overexpression of BCRP in P388 cells, which was observed with the drug alone treatment. This reinforces that Dox/Pluronic formulations prevent formation of MDR phenotype. Our previous work demonstrated that Dox/Pluronic formulation abolished overexpression of Pgp observed upon selection of P388 cells with Dox alone [25]. We have also previously shown considerable changes in the global gene expression profiles in P388 cells and human breast adenocarcinoma MCF7 cells treated with Dox/Pluronic formulation compared to Dox alone [24], [25]. Specifically, in P388 cells the Dox alone treatment affected genes implicated in cell cycle, apoptosis, energy production, cell-to-cell signaling, drug metabolism, stress response, molecular transport and other functions [25]. Some of these genes are directly involved in tumor morphology and tumorigenesis. In contrast Dox/Pluronic prevented alterations of these multiple genes that may be implicated in progression of drug resistance in tumors.

Based on that we hypothesized that SP1049C may alter epigenetic regulation of gene expression compared to drug alone. Epigenetic control of gene expression including genes involved in drug resistance was found to be critical in cancer, carcinogenesis and tumorigenicity [36]. In this work we explored the effects of SP1049C on the profiles of DNA methylation, which is an important regulator of gene transcription. We found herein that all treatment groups (SP polymers, Dox and SP1049C) exhibited strong and distinct from each other patterns of alteration of DNA methylaion compared to the saline control. However, the strongest effect was seen in SP1049C treatment group, which was mostly due to hypomethylation of the gene promoters. Moreover, IPA suggests that the top affected biological functions and canonical pathways in this group relate to cellular function, growth and maintenance, as well as regulation of stem cell differentiation and pluripotency. Albeit there is no simple relationship between DNA methylation and cancer, the finding that SP1049C induced strikingly different and strongest changes in DNA methylation along with decreased tumorigenicity and aggressiveness of P388 cells is significant. Notably, deregulation of DNA methylation patterns of various genes has been connected to tumor cell proliferation, antiapoptosis, neo-angiogenesis, invasive behavior and chemotherapy resistance [51]–[54]. For example it was shown that reducing the methylation by 10% from normal levels was sufficient to induce cancer development in mice [55]. Moreover inhibition of DNA methyltransferases and/or histone deacetylases may induce cancer cell differentiation [56], [57]. Additionally, certain chemotherapeutic drugs induce upregulation of MDR1 gene by hypomethylation of MDR1 gene promoter [58]. On the other hand, inactivation of tumor suppressor genes by hypermethylation has been also reported [51]. However, we would like to point out as a limitation that our findings do not allow to clearly link the described changes in DNA methylation profiles to the observed changes in tumor aggressiveness or tumorigenicity of cancer cells.

Considerable amount of data suggests that small population of CSC is a driving force of tumor initiation, progression, metastasis and drug resistance development. Since CSC display some of the characteristics of MDR cells, like overexpression of drug efflux transporters, we hypothesized that Dox/Pluronic formulation can also be effective against CSC. One of the main characteristics of CSC is overexpression of specific biomarkers. The markers can be different depending on the cancer type and species. The present study has clearly shown that SP1049C effectively depletes the tumorigenic CD133^+^ cell subpopulation upon *in vivo* selection of ascitic P388 leukemia cells. Previously, CD133^+^ cells of different origins were shown to be highly tumorigenic *in vivo* [7], as well as radio- [59] and chemoresistant [60]. The downregulation of CD133 in melanoma cells resulted in the reduction of cell growth, motility, ability to form spheroids and metastatic potential [61]. The reduction in cell growth was proportional to the extent of CD133 downregulation [61]. However, in some cancers CD133^+^ cells did not posses major stem cell properties and did not appear to be linked to tumorigenic cell populations [39]. Therefore, we have confirmed in our model that CD133^+^ cells have functional drug efflux system and are more resistant than CD133^−^ or unsorted cells. We have also shown that these cells have higher tumorigenicity *in vivo* and higher colony formation potential *in vitro*. Thus, it is likely that SP1049C can sensitize CD133^+^ similar to MDR cells reported previously [22]. Moreover, we have shown here that both Dox and SP1049C treatments also deplete tumorigenic ALDH^+^ cell subpopulation compared to saline and polymers alone controls. We have further evaluated the effect of different treatments on CD34^+^/CD38^−^ cell subpopulation. Albeit initially, in Passage 1, both Dox and SP1049C treatments appeared to deplete this subpopulation, all differences with the saline control disappeared by Passage 4. CD34^+^/CD38^−^ phenotype is linked to CSC in human acute myeloid leukemia [3], [43]. However, in our model the CD34^+^/CD38^−^ P388 cells did not display increased resistance or colony-forming potential. Appropriate mouse phenotype for the leukemia stem cells can be explored in the future studies. At the same time we would like to point out a limitation of the characterization of CSC using biomarkers in our work. According to CSC model CSC make only a few percent of the total number of cells, while the populations described above comprise 15% and in some cases even 40% of all cells. This clearly indicates that these populations are not pure and cannot be represented only by CSC. Increased tumorigenicity and drug resistance of these cell populations may suggest that they are enriched with CSC and their early progenies, which may possess similar properties like drug resistance or colony formation potential. Therefore, future studies should focus on possible effects of SP1049C and similar block copolymer-based formulations on the CSC in cancers of various origins, including primary patient tumor samples.

The ability of Pluronic/Dox formulation to deplete drug resistant tumorigenic cell subpopulations such as CD133^+^ cells, which express functional drug efflux system, is generally consistent with the known activities of this formulation against MDR cells. Pluronics were shown to interact with cell membranes where they decrease the membrane microviscosity, and inhibit ATPase activity and drug efflux function of Pgp [62], [63]. Moreover, in contrast to various conventional MDR sensitizers their activity is not restricted to inhibition of Pgp, as they were shown to display complex and profound effects on MDR cancer cells. In particular, Pluronics transport into cells, reach mitochondria, depolarize mitochondrial membrane and inhibit mitochondria respiratory chain [63]. These effects are accompanied with ATP depletion, release of mitochondrial cytochrome c and increased production of reactive oxygen species in MDR cells [63]. As a result Pluronics not only facilitate the entry of the drug into the MDR cells but also enhance proapoptotic signaling and decrease antiapoptotic defense of these cells in response to the drug [64]. Interestingly our data suggest that SP polymers alone can also decrease tumorigenicity of cancer cells upon long-term treatment in vivo such as for 7 passages. This may be consistent with their ability to affect metabolism in drug resistant cancer cells, and exhibit cytotoxic effects upon long-term term treatments of these cells in vitro [63], [65]. Nevertheless, the cytotoxicity of Dox/Pluronic formulations in vitro and the anti-tumor effects in vivo greatly surpass these of the polymers alone. At the same time, Dox/Pluronic formulations are not more toxic with respect to non-MDR cells than the drug alone, because the mitochondrial effects of the copolymer are restricted to MDR cell phenotype. It is possible that similar phenotype selectivity of Dox/Pluronic formulations also exists in the case of tumorigenic cells (including CSC) at least those that display the MDR phenotype.

## Conclusions

Altogether, it is for the first time we report that SP1049C therapy decreases the tumorigenicity and aggressiveness of cancer cells in vivo, suppresses the Wnt-β-catenin signaling activation and BCRP overexpression, differentially alters the DNA methylation profiles, as well as depletes the tumorigenic cell subpopulations in murine leukemia model. Our previously work reported activities of SP1049C and similar formulations in selected and transfected MDR cell lines and led to development of SP1049C as a drug candidate that is currently evaluated clinically. Thus our report has considerable advances over prior publications and suggests that SP1049C may have broader spectrum of action that was initially thought, especially in leukemia and breast cancer, where this polymeric micelle drug was already shown to prevent tumor escape during chemotherapy *in vitro* and *in vivo.* Future studies should use different and additional models to reinforce our findings and demonstrate their relevance to human disease and therapy. However, we believe that the information provided in this manuscript is thought provoking and should be disseminated among pharmaceutical and cancer researchers to promote and facilitate further investigations in this area.

## Supporting Information

Figure S1 **(a)** Tumor images isolated at day 12 from tumor aggressiveness study, shown in Figure 2c. **(b)** Rate of s.c. tumor growth of P388 ascitic cells after different chemotherapy treatment regimens (5×10^5^ cells/mice from Passage 4. Tumor volumes comparisons are presented for day 14 data point; the comparisons were made using Student’s t-test. *p<0.05, **p<0.01. **(c)** Lifespan of animals from tumor aggressiveness study, shown in Figure 2d.
(DOCX)

Figure S2 Top five biological functions (a, b) and canonical pathways (c, d) identifyed by IPA. The genes that experienced >20-fold change in methylation after specific treatment (SP polymers, Dox and SP1049C from Passages 1 (**P1**) and 4 (**P4**)) compared to saline treatment from corresponding passage were identified analyzed using IPA (Ingenuity Systems, CA, USA). The yellow line represents the threshold of –log*P* greater than 1.3.
(DOCX)

Figure S3 Effect of SP1049C on *in vitro* cytotoxicity of Dox. ALDH^−^ (**a**) and ALDH^+^(**b**) cells were isolated from passage 5 saline treated mice using FACS. Ascites cells were collected, washed with ACK buffer and analyzed for ALDH activity using ALDEFLUOR assay according to manufacturers protocol. Dox cytotoxicity was evaluated in the presence or absence of 0.001% SP1049C after 6 days of incubation.
(DOCX)

Figure S4 CD34^+^/CD38^−^ cell subpopulation in ascites during in vivo selection. Ascite cells were collected, washed with ACK buffer and analyzed for CD34 and CD38 expression by FACS. **(a)** Number of colonies formed by unsorted and CD34^+^/CD38^−^ cells isolated from Passage 4 saline treated mice (100 cells seeded/well in 6-well plates, methyl cellulose media, assayed on day 10–14); **(b)** fractions of CD34^+^/CD38^−^ cells after different chemotherapy regimens and Passages. Treatments: 1) saline, 2) SP polymers alone (0.225 mg/kg), 3) Dox (2.5 mg/kg) or 4) SP1049C (2.5 mg/kg Dox, 0.225 mg/kg polymer mixture). ****p<0.0001, n.s. - not significant.
(DOCX)

Table S1 Tumor formation frequency of the cells, isolated from passage 1 (P1) and passage 6 (P6) animals. Treatments: 1) saline, 2) polymers alone (0.225 mg/kg), 3) Dox (2.5 mg/kg) or 4) SP1049C (2.5 mg/kg Dox, 0.225 mg/kg polymer mixture).
(DOCX)

Table S2 Dox cytotoxicity in P388 unsorted ascitic cells and CD34^+^/CD38^−^ cells isolated from Passage 4 saline treated mice using magnetic sorting. Dox cytotoxicity was evaluated after 48 h incubation.
(DOCX)

Table S3 Significantly methylated and demethylated genes. Gene promoters, that have experienced >20 fold change in methylation compared to saline control from corresponding passage in SP polymers alone, Dox alone and SP1049C groups from passages 1 and 4. 20 corresponds to >20 times hypermethylation of gene promoter compared to control, −20 corresponds to >20 times demethylation of gene promoter compared to control. 0 corresponds to <20 times changes methylation of gene promoter.
(XLSX)
