# Supplementary figures and images for: Effect of Doxorubicin/Pluronic SP1049C on Tumorigenicity, Aggressiveness, DNA Methylation and Stem Cell Markers in Murine Leukemia
Source: PLoS One. 2013 Aug 19;8(8):e72238. doi: 10.1371/journal.pone.0072238 (PMC3747131; doi:10.1371/journal.pone.0072238)

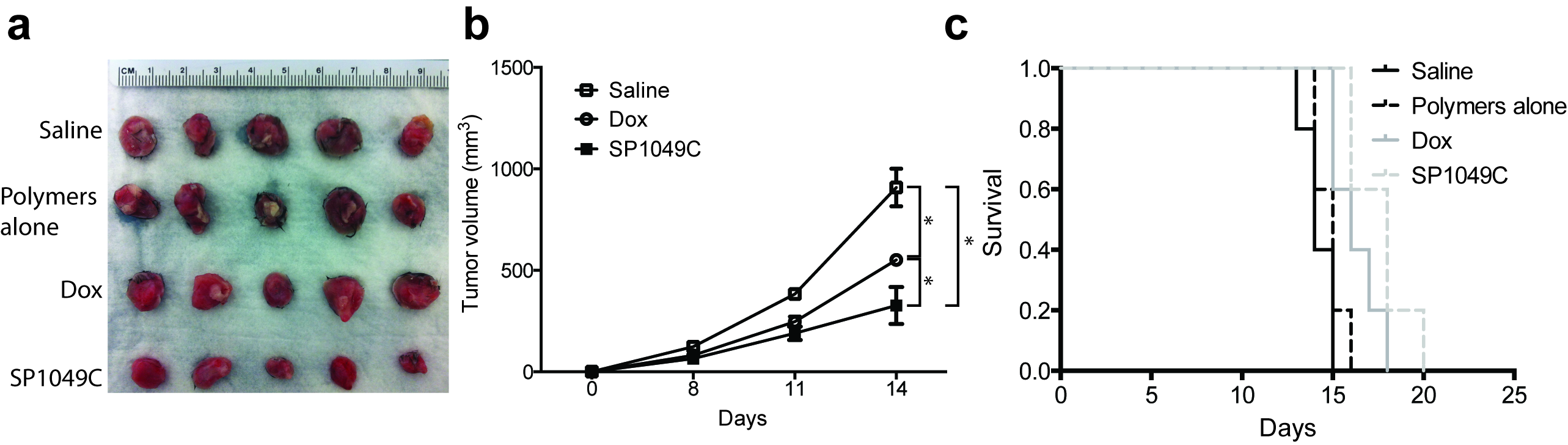

Supplement: Figure S1 — (a) Tumor images isolated at day 12 from tumor aggressiveness study, shown in Figure 2c. (b) Rate of s.c. tumor growth of P388 ascitic cells after different chemotherapy treatment regimens (5×10^5^ cells/mice from Passage 4. Tumor volumes comparisons are presented for day 14 data point; the comparisons were made using Student’s t-test. *p<0.05, **p<0.01. (c) Lifespan of animals from tumor aggressiveness study, shown in Figure 2d. (DOCX) [file pone.0072238.s001.docx]

**
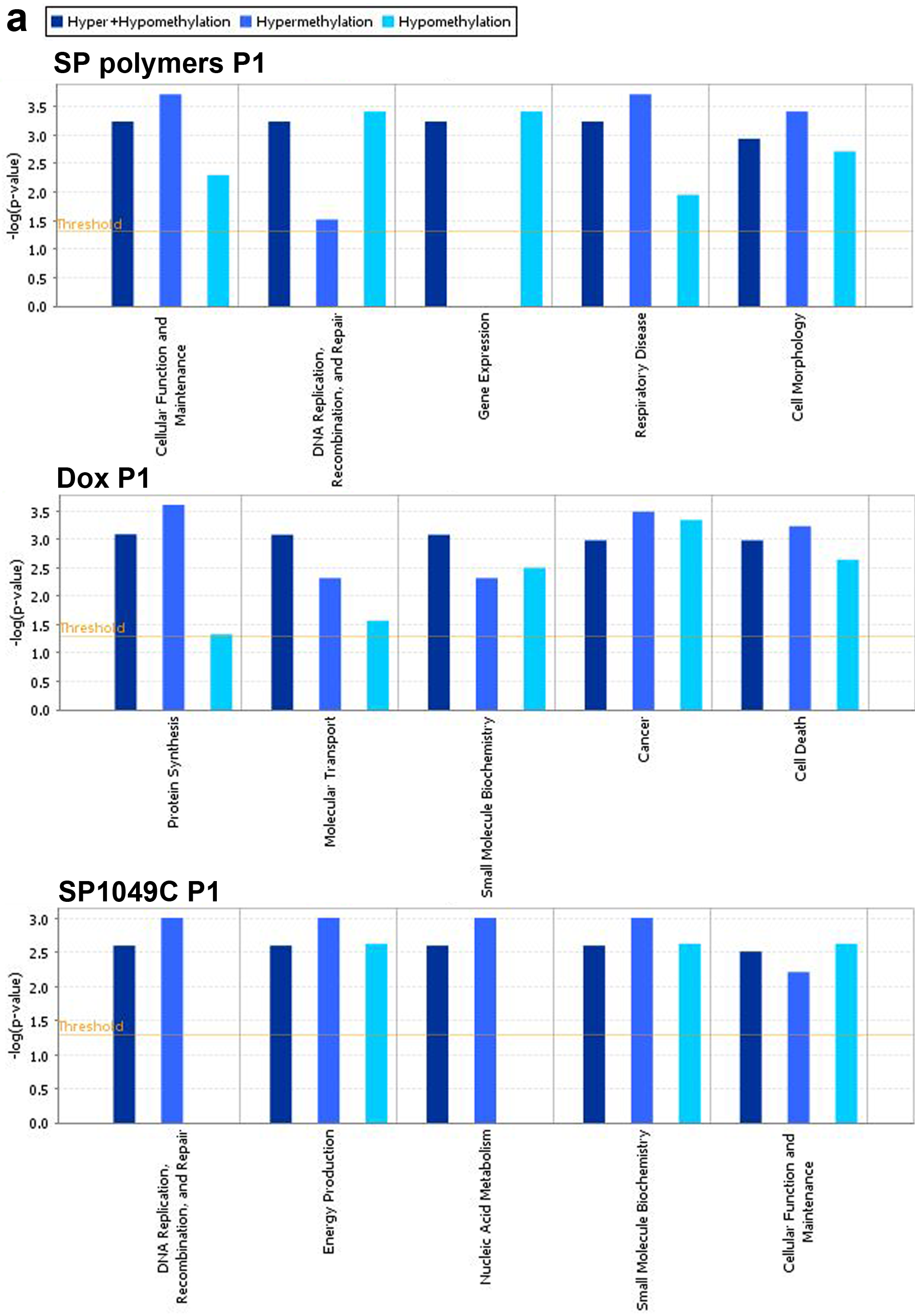
**

**
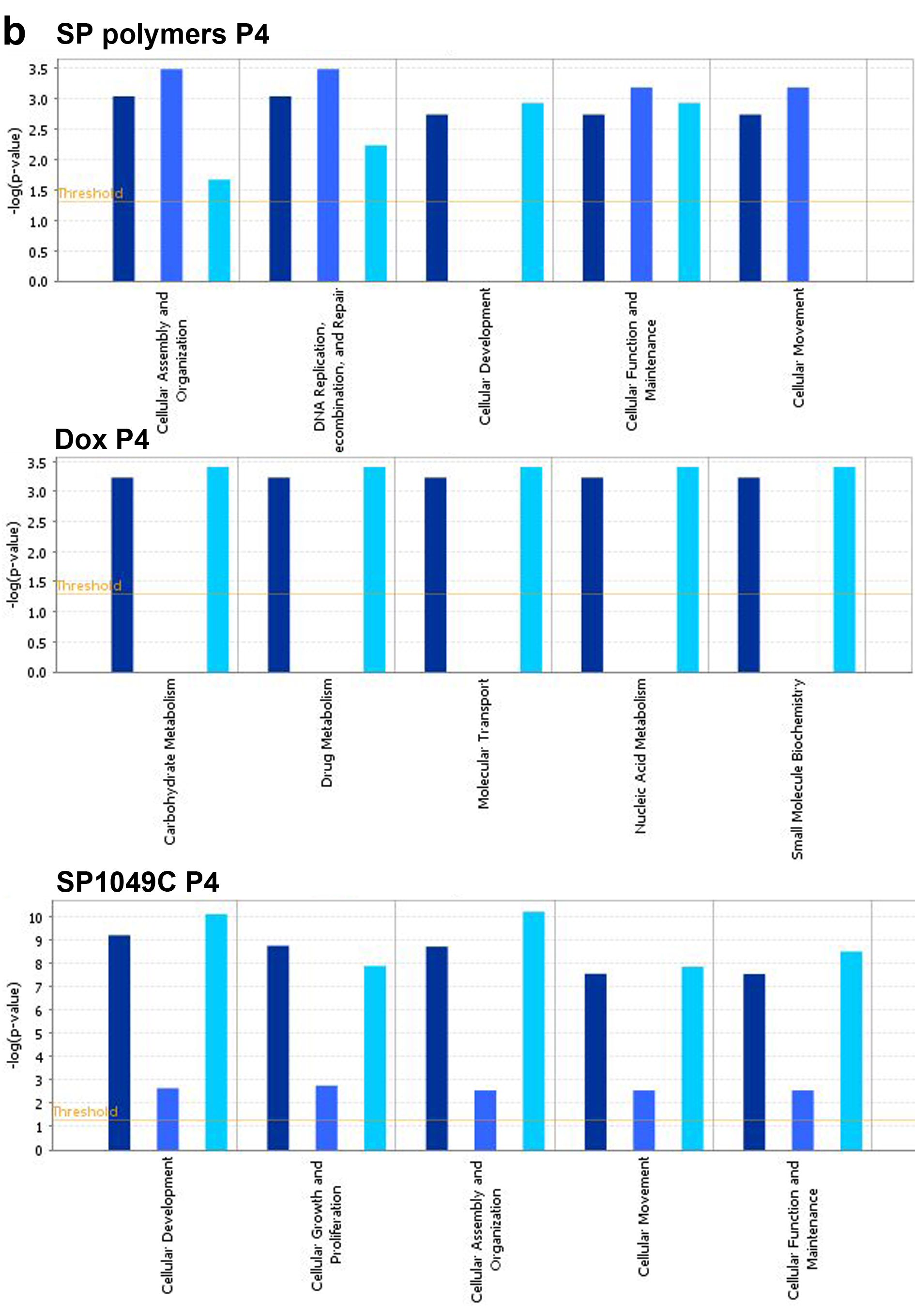
**

**
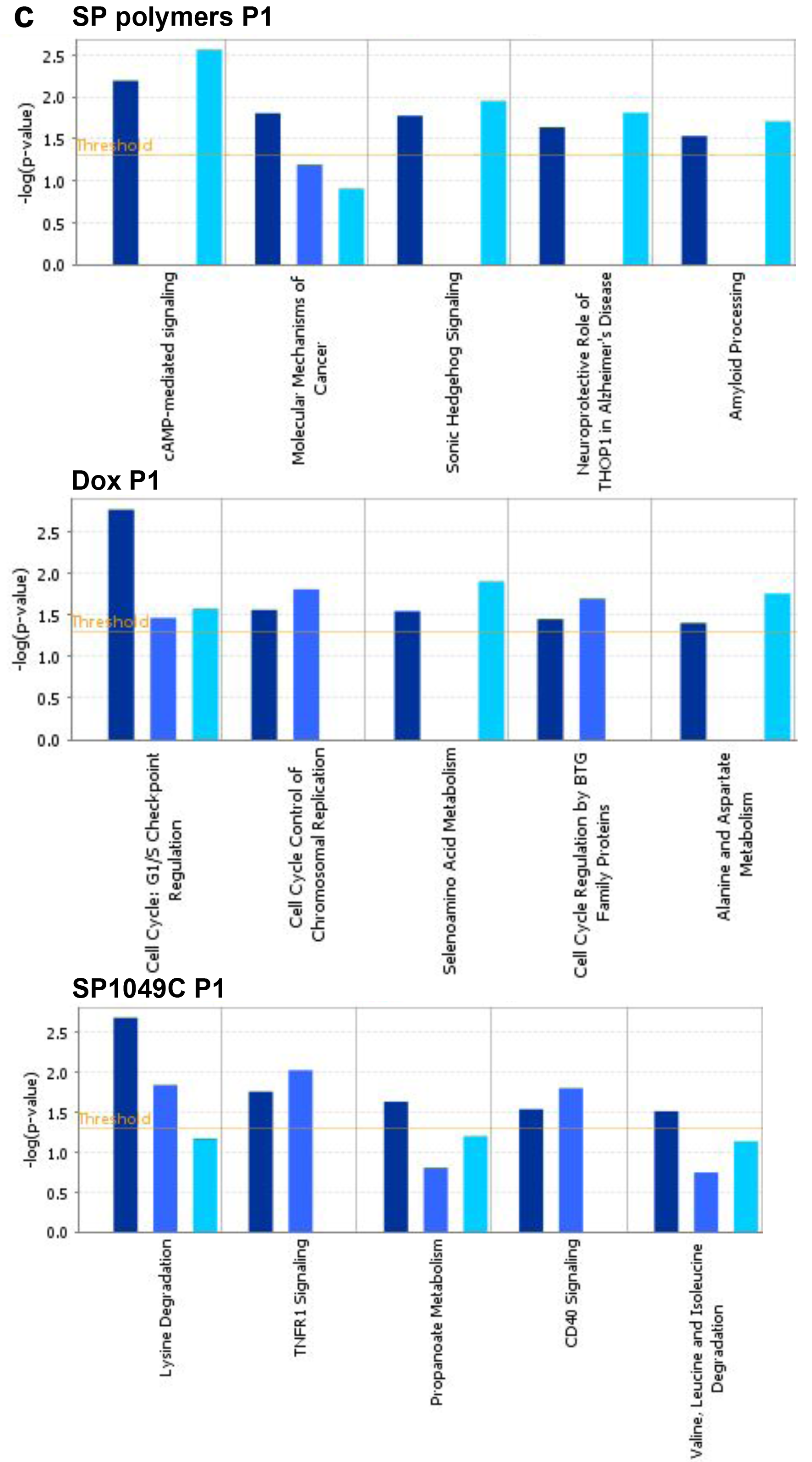
**

**
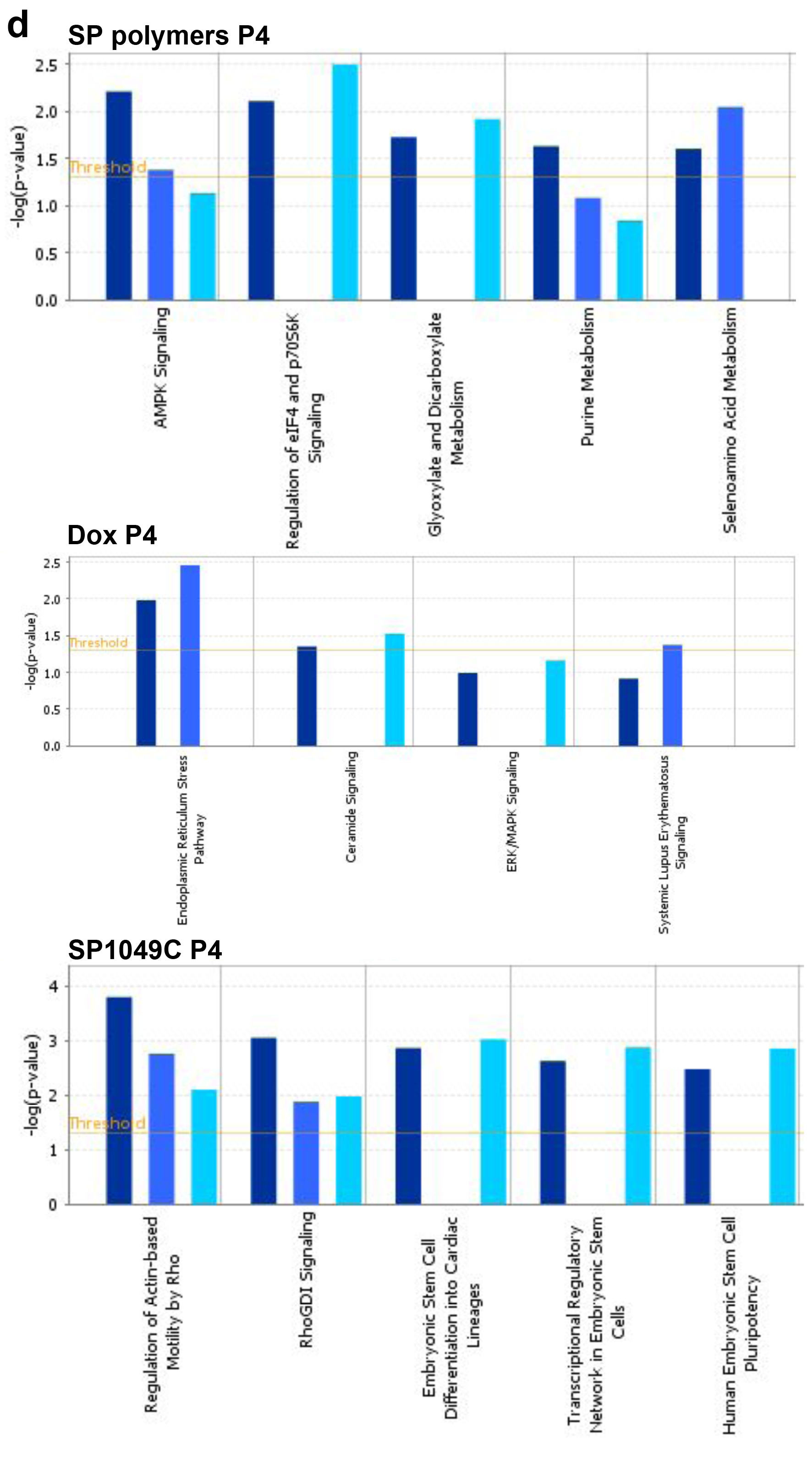
**

Supplement: Figure S2 — Top five biological functions (a, b) and canonical pathways (c, d) identifyed by IPA. The genes that experienced >20-fold change in methylation after specific treatment (SP polymers, Dox and SP1049C from Passages 1 (P1) and 4 (P4)) compared to saline treatment from corresponding passage were identified analyzed using IPA (Ingenuity Systems, CA, USA). The yellow line represents the threshold of –logP greater than 1.3. (DOCX) [file pone.0072238.s002.docx]

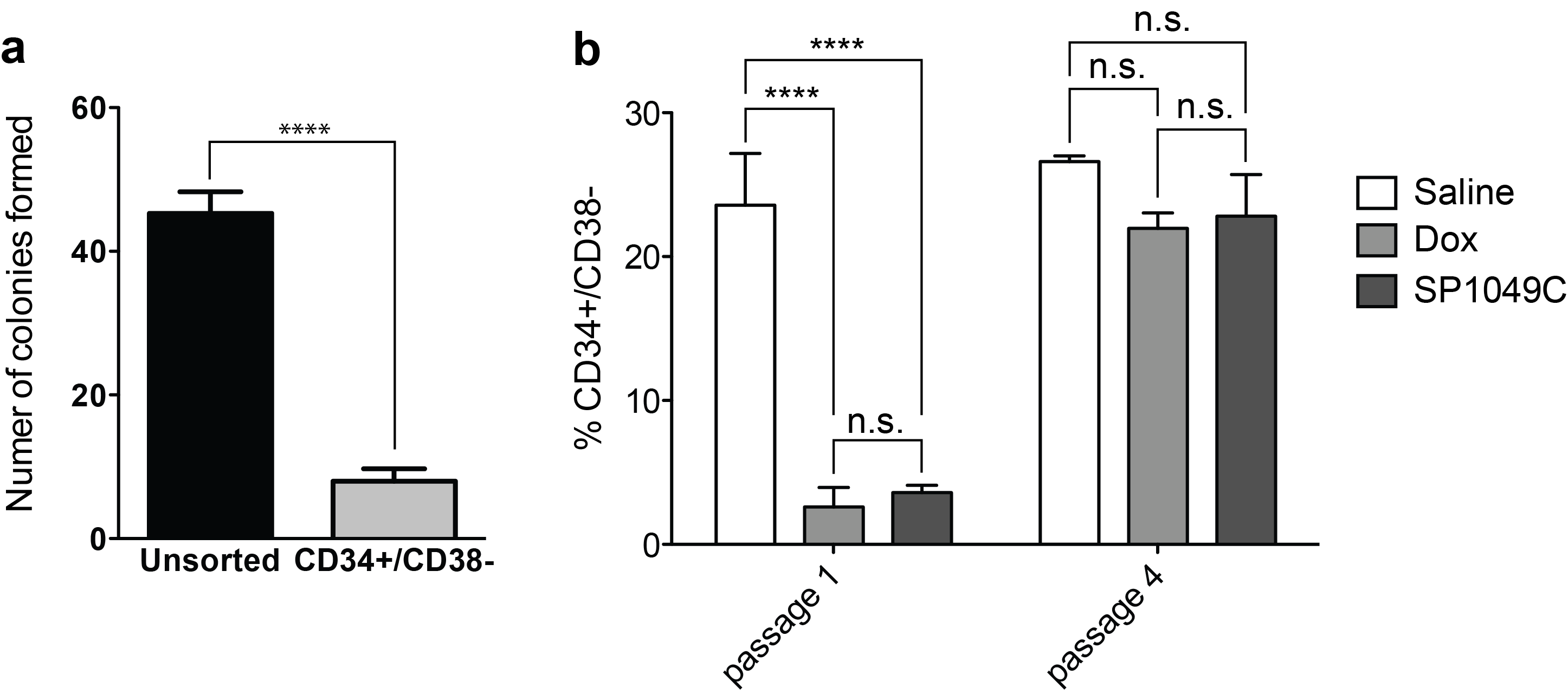

Supplement: Figure S4 — CD34+/CD38− cell subpopulation in ascites during in vivo selection. Ascite cells were collected, washed with ACK buffer and analyzed for CD34 and CD38 expression by FACS. (a) Number of colonies formed by unsorted and CD34+/CD38− cells isolated from Passage 4 saline treated mice (100 cells seeded/well in 6-well plates, methyl cellulose media, assayed on day 10–14); (b) fractions of CD34+/CD38− cells after different chemotherapy regimens and Passages. Treatments: 1) saline, 2) SP polymers alone (0.225 mg/kg), 3) Dox (2.5 mg/kg) or 4) SP1049C (2.5 mg/kg Dox, 0.225 mg/kg polymer mixture). ****p<0.0001, n.s. - not significant. (DOCX) [file pone.0072238.s004.docx]
