# Supplementary material for: Effect of Doxorubicin/Pluronic SP1049C on Tumorigenicity, Aggressiveness, DNA Methylation and Stem Cell Markers in Murine Leukemia
Source: PLoS One. 2013 Aug 19;8(8):e72238. doi: 10.1371/journal.pone.0072238 (PMC3747131; doi:10.1371/journal.pone.0072238)
Supplement: Table S1 — Tumor formation frequency of the cells, isolated from passage 1 (P1) and passage 6 (P6) animals. Treatments: 1) saline, 2) polymers alone (0.225 mg/kg), 3) Dox (2.5 mg/kg) or 4) SP1049C (2.5 mg/kg Dox, 0.225 mg/kg polymer mixture). (DOCX) [file pone.0072238.s005.docx]

| **Cell dose** | **Saline** | | **Dox** | | **Sp1049C** | |
| --- | --- | --- | --- | --- | --- | --- |
|  | **P1** | **P6** | **P1** | **P6** | **P1** | **P6** |
| 5000 | 4/4 | 5/5 | 4/4 | 5/5 | 4/4 | 3/5 |
