# Supplementary material for: Effect of Doxorubicin/Pluronic SP1049C on Tumorigenicity, Aggressiveness, DNA Methylation and Stem Cell Markers in Murine Leukemia
Source: PLoS One. 2013 Aug 19;8(8):e72238. doi: 10.1371/journal.pone.0072238 (PMC3747131; doi:10.1371/journal.pone.0072238)
Supplement: Table S2 — Dox cytotoxicity in P388 unsorted ascitic cells and CD34+/CD38− cells isolated from Passage 4 saline treated mice using magnetic sorting. Dox cytotoxicity was evaluated after 48 h incubation. (DOCX) [file pone.0072238.s006.docx]

| **Treatment** | **Dox IC_50_, ng/mL** | |
| --- | --- | --- |
|  | **Unsorted** | **CD34^+^/CD38^-^** |
| Dox | 17.50 | 16.27 |
