# Supplementary material for: Effect of Doxorubicin/Pluronic SP1049C on Tumorigenicity, Aggressiveness, DNA Methylation and Stem Cell Markers in Murine Leukemia
Source: PLoS One. 2013 Aug 19;8(8):e72238. doi: 10.1371/journal.pone.0072238 (PMC3747131; doi:10.1371/journal.pone.0072238)
Supplement: Supplementary file 4 [file pone.0072238.s003.docx]

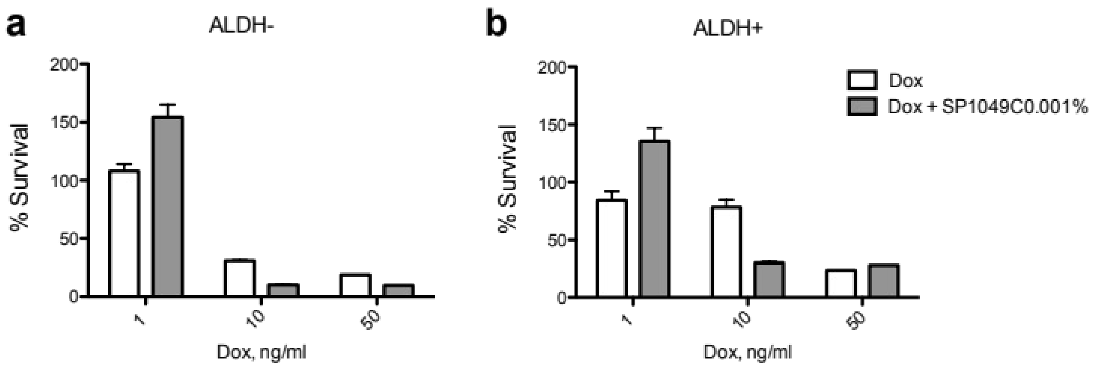
a
ALDH-
200
150
100
50
0
% Survival
1
10
50
Dox, ng/ml
b
ALDH+
200
150
100
50
0
% Survival
1
10
50
Dox, ng/ml
Dox
Dox + SP1049C0.001%
